# An Unusual Presentation of Novel Missense Variant in *PAX6* Gene: NM_000280.4:c.341A>G, p.(Asn114Ser)

**DOI:** 10.3390/cimb46010008

**Published:** 2023-12-22

**Authors:** Tatyana A. Vasilyeva, Natella V. Sukhanova, Olga V. Khalanskaya, Andrey V. Marakhonov, Nikolai S. Prokhorov, Vitaly V. Kadyshev, Nikolay A. Skryabin, Sergey I. Kutsev, Rena A. Zinchenko

**Affiliations:** 1 Research Centre for Medical Genetics, 115522 Moscow, Russia; vasilyeva_debrie@mail.ru (T.A.V.); natelasukhanova@gmail.com (N.V.S.); o.halanskaya@mail.ru (O.V.K.); vvh.kad@gmail.com (V.V.K.); kutsev@mail.ru (S.I.K.); renazinchenko@mail.ru (R.A.Z.); 2 Department of Molecular and Cellular Biochemistry, Indiana University, Bloomington, IN 47405, USA; niprokho@iu.edu; 3 Research Institute of Medical Genetics, Tomsk National Research Medical Center of the Russian Academy of Sciences, 634050 Tomsk, Russia; nikolay.skyabin@medgenetics.ru

**Keywords:** retina coloboma, iris stromal defect, *PAX6* missense variant, gain-of-function mutation

## Abstract

This study investigates a unique and complex eye phenotype characterized by minimal iris defects, foveal hypoplasia, optic nerve coloboma, and severe posterior segment damage. Through genetic analysis and bioinformatic tools, a specific nonsynonymous substitution, p.(Asn114Ser), within the *PAX6* gene’s paired domain is identified. Although this substitution is not in direct contact with DNA, its predicted stabilizing effect on the protein structure challenges the traditional understanding of *PAX6* mutations, suggesting a gain-of-function mechanism. Contrary to classical loss-of-function effects, this gain-of-function hypothesis aligns with research demonstrating PAX6’s dosage sensitivity. Gain-of-function mutations, though less common, can lead to diverse phenotypes distinct from aniridia. Our findings emphasize *PAX6*’s multifaceted influence on ocular phenotypes and the importance of genetic variations. We contribute a new perspective on *PAX6* mutations by suggesting a potential gain-of-function mechanism and showcasing the complexities of ocular development. This study sheds light on the intricate interplay of the genetic alterations and regulatory mechanisms underlying complex eye phenotypes. Further research, validation, and collaboration are crucial to unravel the nuanced interactions shaping ocular health and development.

## 1. Introduction

The *PAX6* gene encodes a transcription factor that plays a critical role in eye development and maintenance. Mutations in the *PAX6* gene can lead to a range of eye abnormalities, including aniridia, cataracts, and corneal opacities, among others [1,2]. *PAX6* loss-of-function mutations are mainly associated with a congenital aniridia phenotype, which presents complex effects on almost all eye structures in its anterior and posterior segments, including the iris, cornea, lens, anterior chamber angle, retina, and optic nerve. *PAX6* missense mutations predominantly lead to non-aniridia phenotypes characterized by distinct effects on one of the abovementioned eye structures, which could include either severe macula hypoplasia with achromatopsia, severe microphtalmia, optic nerve coloboma, or a morning glory disc anomaly [2,3].

One reason that different missense mutations in the *PAX6* gene can produce different phenotypes is that the location and nature of the mutation can affect the function of the resulting protein. Missense mutations are changes in a single nucleotide that result in a different amino acid being incorporated into the protein. Depending on where the missense mutation occurs within the protein sequence, it can affect the protein’s ability to bind DNA, interact with other proteins, or perform other critical functions [4].

Additionally, the *PAX6* gene is subject to complex regulatory mechanisms that can further influence its function. These regulatory mechanisms can include factors such as alternative splicing, post-translational modifications, and interactions with other regulatory proteins. Different missense mutations may affect these regulatory mechanisms in different ways, leading to different phenotypic outcomes [5].

Finally, other genetic and environmental factors can also contribute to the phenotypic variability observed in individuals with *PAX6* mutations. For example, mutations in other genes that interact with or regulate *PAX6* can modify the effect of *PAX6* mutations [6,7]. Additionally, environmental factors such as exposure to toxins or infections during development can interact with genetic factors to affect eye development and lead to phenotypic variability [8].

The mechanisms of missense mutations in the *PAX6* gene are still not sufficiently understood. There are several general ideas based on the evaluation of the binding activity of this transcription factor with adjustable targets. However, several issues remain: the first is the extreme rarity of missense mutations, and the second is that mainly non-aniridia phenotypes are observed patients with such mutations [9].

Here, we present the unusual clinical phenotype of a patient with optic nerve coloboma and macular hypoplasia that is explained by a novel missense mutation in the *PAX6* gene.

## 2. Case Presentation

### Materials and Methods

A full eye examination was carried out on the patient. This included ophthalmoscopy (fundus examination), electrophysiological examination, computer perimetry, ultrasound examination (B-scan), visometry, autorefractometry, pneumotonometry, biometry, pachymetry, and biomicroscopy.

Genomic DNA was isolated from peripheral blood using a Promega^®^ kit according to the manufacturer’s recommendations. Copy number variations in the 11p13 locus were analyzed by multiplex ligase-dependent probe amplification (MLPA) analysis, and intragenic *PAX6* sequence variants were screened by Sanger sequencing, as mentioned earlier [10].

ColabFold v1.5.3, an implementation of Alphafold2, was used to generate computed structure models (CSMs) of WT PAX6 and its N114S variant [11,12]. CSMs were visualized with UCSF ChimeraX v1.7 [13].

Patient S, a 28-year-old married woman, sought consultation at the Research Centre for Medical Genetics (RCMG) in 2023 as she planned to have a child. Her visit was prompted by a complex range of eye defects that she had experienced. Additionally, she complained of poor far and near vision in her right eye and relied on a prosthesis for her left eye.

In her medical history, nystagmus appeared at 6 months of age, and her left eye had been blind since birth. Despite seeking help from specialized ophthalmologic clinics, no treatment or correction was offered. A local ophthalmologist diagnosed her with congenital pathology of the visual organ, accompanied by horizontal nystagmus in both eyes and subatrophy of the left eyeball. The patient denied any aggravated family history, and her physical and intellectual development was in line with her age. There were no chronic diseases reported.

During the general examination, blepharophimosis was evident, and she tilted her head back to improve visual interaction.

The ophthalmologic examination revealed the following findings (Table 1).

**Figure 1:**
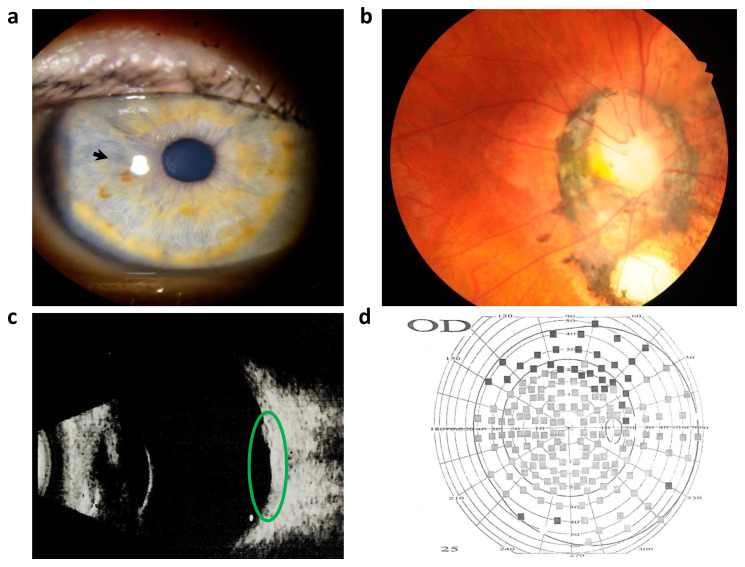
Ophthalmologic examination of the right eye. (**a**) Slit lamp examination depicted a radial elliptical anterior iris stromal defect (black arrow). (**b**) Retinal imaging conducted by a fundus camera displayed a pale pink, rounded optic disc with clear borders. An optic disc coloboma measuring half of the optic disc’s size was observed, accompanied by a notable deposit of black pigment. The macular zone exhibited an indistinct boundary. (**c**) Ultrasound B-scan of the right eye, focusing on the congenital optic disc coloboma (green ellipse), revealed clear optical media, membranes with adherent properties, and no presence of additional inclusions. (**d**) The result of computerized perimetry conducted on the right eye (OD) revealed narrowing of the peripheral borders of the visual field and relative scotoma up to 10 degrees from the fixation point.

Right eye (OD) visometry showed uncorrected visual acuity of −0.1. Autorefractometry showed sph −0.75, cyl −2.75 ax 101 and a corneal thickness at pachymetry of 562 µm. Pneumotonometry measured the intraocular pressure at −15 mmHg, and biometry indicated an axial length of 21.01 mm. Biomicroscopy revealed a calm conjunctiva and transparent cornea. The anterior chamber depth was within the normal range. In the upper outer quadrant, the iris was structurally changed and slightly hypopigmented, and a radial elliptical anterior iris stromal defect was visualized (Figure 1a), while the crystalline lens was transparent.

Left eye (OS) visometry resulted in uncorrected visual acuity of −0. Biomicroscopy showed subatrophy of the eyeball with a clear conjunctiva. The cornea was reduced in size and presented an old scar with vascularization. The anterior chamber was shallow and irregular, and the pupil had an irregular shape. Ophthalmoscopy was not possible for the left eye.

Further examination of the right eye (OD) using ophthalmoscopy revealed an enlarged optic nerve disc with a deep funnel-shaped excavation, clearly demarcated borders, and surrounded by a ring of damaged hyperpigmented choroid and retina. In the parapapillary area, persistent hyaloid remnants were observed (Figure 1b). Retinal vessels appeared narrowed but with a uniform caliber. The macular zone lacked differentiation, and no reflexes were evoked. OS was not ophthalmoscopically detectable. In the electrophysiologic study, regarding OD, the electrical sensitivity thresholds were within the normal range, measuring 130 µA. Electrical lability was also normal, with a frequency of 43 Hz (normal range: 40–50 Hz). For the left eye (OS), electrical sensitivity thresholds were within the normal range (40–80 µA), and no phosphene response was evoked. In the B-scan of the right eye (OD), a defect was observed in the posterior pole of the eye, accompanied by the slight enlargement of the proximal segment of the optic nerve where it contacted the sclera (Figure 1c). During the computerized perimetry of the right eye (OD), the boundaries remained unchanged. Paracentral and peripheral focal scotomas were detected, particularly in the upper parts of the visual field. The blind spot area showed no distinctive features, exhibiting a suprathreshold brightness of 35 dB and a complete loss of fixation at 100% (Figure 1d).

Further assessment and evaluation were performed to provide comprehensive care and assist the patient in her journey towards parenthood.

**Figure 2:**
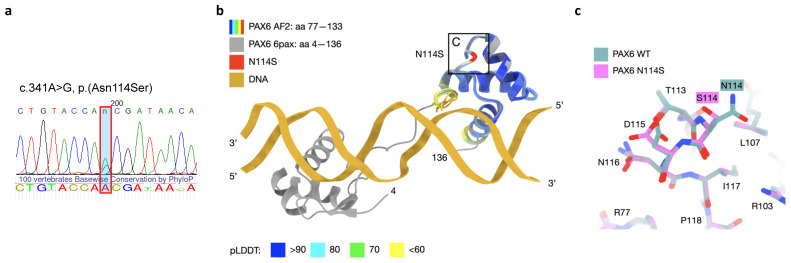
Genetic examination of the patient. (**a**) Sanger sequencing revealed NM_000280.4(*PAX6*):c.341A>G variant in a heterozygous state; 100 vertebrate conservation score by PhyloP is depicted under the sequenogram. (**b**) Modeling of the substitution in the paired domain of the PAX6 protein. The five best Alphafold2 computed structure models (CSMs) of WT PAX6 and the five best AF2 CSMs of the N114S variant are superimposed with the previously experimentally determined crystal structure of PAX6 (PDB accession number 6pax) [20]. The CSMs are colored according to the confidence of prediction (pLDDT score). (**c**) Superposition of the best CSMs of WT PAX6 and N114S variant in proximity to the mutation, illustrating moderate local changes in the structure. The transition is predicted to preserve intra-molecular interactions.

Molecular genetic analysis of the *PAX6* gene was carried out using a published algorithm [14]. During Sanger sequencing, a novel single nucleotide variant, NM_000280.4:c.341A>G, was identified in a heterozygous state in exon 6 of the *PAX6* gene. This variant results in a missense substitution of the highly conservative amino acid residue p.(Asn114Ser) within the paired domain (RED subdomain) of the PAX6 protein (Figure 2a). The identified variant was found on one allele out of a total of 251,484 alleles in the publicly available population database gnomAD v2.1.1 [15]. However, it was not present in other databases such as Kaviar and RuSeq [16]. Classical pathogenicity predictors, including SIFT, Polyphen 2, and MutationTaster, give varying predictions, classifying the variant as either moderately damaging, uncertain, or benign. On the other hand, meta-score predictors suggest that the variant is likely to be damaging (see Supplementary Table S1). Segregation analysis could not be performed in this case due to the unavailability of the patient’s parents for analysis. The absence of parental genetic information hindered the ability to determine whether the identified novel variant was inherited from one of the parents or occurred de novo in the patient.

Considering the available information, the variant is currently categorized as a variant of unknown clinical significance. Its potential relationship with the patient’s phenotype is yet to be fully understood.

The functional consequences of the identified missense substitution were further analyzed using several bioinformatic tools. The FASPR tool [17] allowed the visualization of the altered residue in the turn region between the second and third helices in the RED subdomain of the PAX6 protein (AF-P26367-F1) [11] (Figure 2b). DynaMut2 [18] predicted a stability change ΔΔG^Stability^ of 0.42 kcal/mol, suggesting that the variant may have a stabilizing effect on the protein. This means that the substitution of the amino acid residue p.(Asn114Ser) in the PAX6 protein may lead to a more stable protein structure compared to the wild type. The MIZTLI resource indicated that the relative solvent accessibility of the substituted residue is not predicted to change [19]. Solvent accessibility refers to the accessibility of an amino acid residue to the surrounding aqueous environment, and if it remains unchanged, it may suggest that the variant does not significantly alter the interaction of the residue with its environment (Figure 2c). This visualization may provide insights into how the missense substitution affects the local structure and potential interactions of the PAX6 protein.

## 3. Discussion

A 28-year-old female patient sought genetic counseling from the experts at the RCMG due to a multifaceted eye phenotype characterized by the coexistence of optic nerve coloboma, foveal hypoplasia, and microphthalmia. This complex presentation prompted the initiation of target sequencing for the *PAX6* gene.

During the genetic analysis, a single nucleotide variant was detected in a heterozygous state, specifically NM_000280.4(*PAX6*):c.341A>G. This variant is situated within exon 6 of the *PAX6* gene and results in a nonsynonymous substitution, p.(Asn114Ser). Notably, this substitution occurs within the sequence of the RED subdomain of the paired domain within the PAX6 protein. The *PAX6* gene serves as a pivotal transcription factor that plays a crucial role in regulating the embryonic development of the eye. This gene’s expression occurs within various cell lines found in the eye, following a precisely defined pattern that is specific to the spatial, temporal, dosage, and pathway considerations inherent to each tissue.

Loss-of-function mutations in the *PAX6* gene typically result in the well-recognized classical phenotype of aniridia, which is characterized by the absence of the iris and often accompanied by foveal hypoplasia. This phenotype is frequently coupled with additional ocular manifestations, such as cataracts, keratopathy, and anomalies affecting the optic nerve and other eye structures. These outcomes are primarily attributed to the haploinsufficiency mechanism, where a single functioning allele of the PAX6 gene fails to produce sufficient protein to ensure proper eye development [21].

In contrast, nonsynonymous substitutions in the *PAX6* gene may exhibit distinct effects compared to the straightforward haploinsufficiency mechanism. Rather than solely relying on the reduction in functional protein levels, these substitutions could trigger alternative and more intricate pathways that affect eye development. The impact of these substitutions might be deferred or delayed, revealing their influence through diverse mechanisms [9,22]. Indeed, nonsynonymous substitutions in the *PAX6* gene have the potential to impact various aspects of protein function and regulation. These substitutions can influence the stability of the protein, alter its folding process, modify its affinity and specificity for regulated target sequences, and interfere with crucial regulatory mechanisms. Interestingly, these nonsynonymous substitutions are frequently associated with phenotypes that extend beyond the classical aniridia presentation. These diverse outcomes encompass conditions such as microphthalmia, congenital cataracts, Peters’ anomaly, isolated foveal hypoplasia, and abnormalities affecting the optic nerve [22]. The breadth of these associated phenotypes underscores the intricate and multifaceted roles that the *PAX6* gene plays during eye development and maintenance [23].

Indeed, specific missense substitutions in the *PAX6* gene can recurrently give rise to distinct clinical phenotypes. For example, the p.Arg128Cys substitution has been identified in unrelated patients and consistently linked to foveal hypoplasia [3]. Researchers, such as Alibés et al., have theorized that substitutions within the paired domain of PAX6, involving residues responsible for DNA contact, tend to result in foveal hypoplasia. This may occur by altering the specificity of target binding [9].

In their efforts to understand the mechanisms underlying the generation of *PAX6* missense mutations linked to optic nerve anomalies, Azuma et al. conducted a functional study. Their findings revealed that these missense substitutions were situated within distinct functional domains of the PAX6 protein. Interestingly, these substitutions did not exert an influence on PAX6’s stability or alter its transcriptional or translational levels. However, the study suggested that these missense substitutions had the potential to impact the regulatory properties of the coregulatory pair PAX6/PAX2 [24]. This implies that while the stability and overall abundance of the PAX6 protein remain unaffected, the intricate interplay between PAX6 and PAX2, two crucial regulators, might be disrupted. This regulatory perturbation in the PAX6/PAX2 coregulatory pair could contribute to the development of optic nerve anomalies observed in affected individuals.

By delving into these functional insights, the study contributes to a deeper understanding of how specific genetic variations within the *PAX6* gene can result in optic nerve anomalies. This research underscores the intricate nature of genetic regulation and interactions, providing valuable knowledge that helps to unravel the complex mechanisms underlying ocular developmental disorders [3].

In our observations, a complex eye phenotype has been identified, characterized by a minimal iris defect, a clear lens, and substantial damage to the posterior segment of the eye. This includes foveal hypoplasia and optic nerve coloboma. Notably, this phenotype is associated with a specific nonsynonymous substitution that occurs within a highly conservative residue located in the PAX6 paired domain: p.(Asn114Ser). Our findings are similar to that described by Sharan et al. [25]. We compared the phenotype descriptions of the two cases (Table 2).

Upon integrating the findings from various bioinformatic tools, it becomes apparent that the identified missense substitution p.(Asn114Ser) in the PAX6 protein may exert a stabilizing influence on the protein’s structure. Importantly, this substitution does not seem to significantly alter the relative solvent accessibility of the affected residue. Considering this stabilizing potential, it is plausible that the substitution might result in a gain-of-function effect.

The notion of a gain-of-function effect suggests that the alteration introduced by the p.(Asn114Ser) substitution could potentially enhance the functional capabilities of the PAX6 protein, rather than impairing it. This scenario contrasts the more commonly observed loss-of-function effects associated with *PAX6* gene mutations [26]. Given the intricate role of PAX6 in eye development and maintenance, such a gain-of-function effect could contribute to the unique and complex eye phenotype that we observed [27].

The *PAX6* gene exhibits sensitivity to dosage alterations, which can lead to detrimental effects caused by both loss-of-function and gain-of-function mutations [28]. It is worth noting that while *PAX6* gain-of-function mutations have the potential to induce a range of effects that differ from the classical aniridia phenotype, they could still have significant implications. For instance, tandem duplications involving the *PAX6* gene on chromosome 11p13 have been identified in patients with distinct phenotypes that deviate from aniridia. Specifically, these duplications have been linked to conditions such as microphthalmia or macular dysfunction [29,30].

These findings highlight the intricate balance that the *PAX6* dosage must maintain for proper eye development. Both loss-of-function and gain-of-function mutations can disturb this balance, resulting in a range of ocular phenotypes. The observation of tandem duplications leading to different eye-related outcomes underscores the diverse and nuanced roles of PAX6 in orchestrating the complex processes that shape ocular development and function.

## 4. Conclusions

In conclusion, our investigation into a patient presenting a complex eye phenotype, characterized by minimal iris defects, foveal hypoplasia, optic nerve coloboma, and severe posterior segment damage, has provided intriguing insights into the role of the *PAX6* gene in ocular development. The identification of a specific nonsynonymous substitution, p.(Asn114Ser), within the highly conservative residue of the *PAX6* paired domain, prompted a comprehensive exploration of its potential impact.

Through a combination of genetic analysis, functional predictions, and bioinformatic tools, we have gained a multifaceted understanding of the potential consequences of this missense substitution. While this residue does not show direct contact with DNA, the substitution’s predicted stabilizing effect on the protein’s structure raises the possibility of a gain-of-function mechanism. This hypothesis challenges the conventional understanding of *PAX6* mutations, which often lead to loss-of-function effects. The complexity of this phenotype suggests that the *PAX6* dosage sensitivity contributes to the intricacies of ocular development.

Our findings align with previous research indicating that *PAX6* dosage alterations can result in both loss-of-function and gain-of-function mutations, with the latter possibly leading to diverse and distinct phenotypes. This underscores the importance of *PAX6* in regulating the delicate balance required for normal eye development and functioning. Tandem duplications involving the PAX6 gene have been associated with variations in eye-related outcomes, further emphasizing the gene’s intricate role in shaping ocular phenotypes.

In summary, this study highlights the complexities of *PAX6*’s involvement in eye development and underscores the significance of genetic variations in shaping diverse ocular phenotypes. While shedding light on the potential mechanisms underlying the observed eye phenotype, our findings also underscore the need for further research, functional validation, and collaborative efforts to unravel the intricate interactions and mechanisms that govern ocular development and health.

Further in-depth studies, both in terms of molecular mechanisms and functional consequences, will be imperative to validate this hypothesis and provide a comprehensive understanding of the impact of the p.(Asn114Ser) substitution on the PAX6 protein and the observed eye phenotype.

## Figures and Tables

**Table 1:** Results of patient’s eye examination.

Eye Structure	Left Eye (Subatrophy)	Right Eye
Visual acuity	0	0.1
Cornea	Reduced in size and presented an old scar with vascularization	Transparent cornea, corneal thickness at pachymetry of 562 µm
Anterior chamber	Shallow and irregular	Depth was within normal range
Iris	Irregular shape of the pupil	Structurally changed in the upper outer quadrant, slightly hypopigmented with radial elliptical anterior iris stromal defects
Lens	n/a *	Transparent
Intraocular pressure	n/a *	15 mm Hg
Optic nerve	Ophthalmoscopy was not possible	Enlarged optic nerve disc with a deep funnel-shaped excavation, clearly demarcated borders, and surrounded by a ring of damaged hyperpigmented choroid and retina, persistent hyaloid remnants
Macular zone	n/a *	Lacks differentiation and no reflex

* n/a—not available.

**Table 2:** Clinical features of ours and a similar patient with missense *PAX6* variant described earlier.

Patient	This Study	Sharan et al. [25]
Age	28 y.o.	11 y.o.
Gender	Female	Female
Visual acuity (Snellen chart)	OD 20/200 OS proectio lucis certa	OD 20/240-20/480 OS 20/240-20/480
Anterior segment	elliptical iris defects, bilateral iris stromal hypoplasia, corectopia	bilateral elliptical anterior stromal iris defects, superonasal corectopia
Posterior segment	enlarged optic nerve disc with a deep funnel-shaped excavation, surrounded by a ring of damaged hyperpigmented choroid and retina, foveal hypoplasia, nystagmus appeared at 6 mo	hypopigmented fundus, severe photophobia, bilateral nystagmus
Other findings	optic nerve coloboma	
*PAX6* mutation (NM_000280.4)	c.341A>G, p.(Asn114Ser).	c.107G>A, p.(Gly36Glu)

## Data Availability

The datasets used and/or analyzed during the current study are available from the corresponding author upon reasonable request.

## Supplementary Materials

The following supporting information can be downloaded at: https://www.mdpi.com/article/10.3390/cimb46010008/s1, Table S1: Bioinformatic predictions of NM_000280.4:c.341A>G effect.
